# Reviewers List

**DOI:** 10.1002/rmb2.12260

**Published:** 2019-01-15

The publication of invaluable papers in the Reproductive Medicine and Biology depends on the prompt, careful review of submitted manuscripts. We would like to thank the Editorial Board members, the Editorial Advisory Board members, and the following experts for reviewing manuscripts from November 1, 2017 to October 31, 2018.

Yasuhisa Araki

Toshiaki Endo

Shinichiro Fukuhara

Atsushi Fukui

Tatsuro Furui

Ryoei Hara

Akiko Hasegawa

Shu Hashimoto

Osamu Hiraike

Yuji Hirao

Kenichiro Hiraoka

Yasushi Hirota

Hideki Igarashi

Hiroshi Ishikawa

Tomonori Ishikawa

Kenta Ishimoto

Junya Ito

Takeshi Iwasa

Akira Iwase

Haruhiko Kanasaki

Kazuhiro Kawamura

Yasushi Kawano

Khaleque Khan

Iwaho Kikuchi

Kazuhiro Kikuchi

Fuminori Kimura

Masaki Kimura

Naoko Kimura

Satoshi Kishigami

Hideyuki Kobayashi

Yoshitomo Kobori

Keiji Kuroda

Izumi Kusuki

Sung Ki Lee

Seiji Mabuchi

Ryo Maekawa

Hirotaka Masuda

Takehiko Matsuyama

Yasuyuki Mio

Mineto Morita

Tetsunori Mukaida

Motoo Nabeta

Masashi Nagano

Koji Nakagawa

Akitoshi Nakashima

Mikiya Nakatsuka

Kaei Nasu

Emiko Nishioka

Toshiaki Noce

Junko Noguchi

Kazuyuki Ohbo

Masato Ohtsuka

Chikara Ohyama

Hidetaka Okada

Osamu Okitsu

Hiroshi Okuno

Kuniaki Ota

Nobuaki Ozawa

Hiroyuki Sasaki

Takuya Sato

Aya Shimizu

Takeshi Shin

Makio Shozu

Tatsuya Suzuki

Masahito Tachibana

Shingo Takada

Seido Takae

Kazumasa Takahashi

Toshifumi Takahashi

Seiji Takashima

Masahiko Takemura

Toshiaki Taketani

Fuminori Taniguchi

Jumpei Terakawa

Tateki Tsutsui

Hiroshi Uchida

Sayaka Uchida

Hideaki Watanabe

Xiaohua Wu

Shunji Yamada

Yuri Yamamoto

Yasuhisa Yamashita

Hiroaki Yoshida

Yasushi Yumura

